# Comparison of functional disabilities, place of death and end-of-life medical expenditures among centenarians and non-centenarians in China: a series of cross-sectional studies

**DOI:** 10.1186/s12877-023-04111-w

**Published:** 2023-06-30

**Authors:** Zhong Li, Ziqin Ding, Panpan Zhao

**Affiliations:** 1 School of Health Policy and Management, Nanjing Medical University, Nanjing, Jiangsu China; 2 The First School of Clinical Medicine, Nanjing Medical University, Nanjing, Jiangsu China; 3 The First Affiliated Hospital, Nanjing Medical University, Nanjing, Jiangsu China

**Keywords:** Functional disabilities, Place of death, Medical expenditures, End-of-life, Centenarians, China

## Abstract

**Background:**

Long-term and end-of-life (EOL) care for older adults has become a global concern due to extended longevity, which is generally accompanied by increased rates of disability. However, differences in the rates of disability in activities of daily living (ADLs), place of death and medical expenditures during the last year of life between centenarians and non-centenarians in China remain unknown. This study aims to fill this research gap to inform policy efforts for the capacity-building of long-term and EOL care for the oldest-old, especially for centenarians in China.

**Methods:**

Data from 20,228 decedents were derived from the 1998–2018 Chinese Longitudinal Healthy Longevity Survey. Weighted logistic and Tobit regression models were used to estimate differences in the prevalence of functional disability, rate of death in hospitals and EOL medical expenditures by age groups among oldest-old individuals.

**Results:**

Of the 20,228 samples, 12,537 oldest-old individuals were female (weighted, 58.6%, hereafter); 3,767 were octogenarians, 8,260 were nonagenarians, and 8,201 were centenarians. After controlling for other covariates, nonagenarians and centenarians experienced a greater prevalence of full dependence (average marginal differences [95% CI]: 2.7% [0%, 5.3%]; 3.8% [0.3%, 7.9%]) and partial dependence (6.9% [3.4%, 10.3%]; 15.1% [10.5%, 19.8%]) but a smaller prevalence of partial independence (-8.9% [-11.6%, -6.2%]; -16.0% [-19.1%, -12.8%]) in ADLs than octogenarians. Nonagenarians and centenarians were less likely to die in hospitals (–3.0% [–4.7%, –1.2%]; –4.3% [–6.3%, –2.2%]). Additionally, nonagenarians and centenarians reported more medical expenditures during the last year of life than octogenarians with no statistically significant differences.

**Conclusion:**

The oldest-old experienced an increased prevalence of full and partial dependence in ADLs with increasing age and reported a decline in the prevalence of full independence. Compared with octogenarians, nonagenarians and centenarians were less likely to die in hospitals. Therefore, future policy efforts are warranted to optimise the service provision of long-term and EOL care by age patterns for the oldest-old population in China.

**Supplementary Information:**

The online version contains supplementary material available at 10.1186/s12877-023-04111-w.

## Background

Globally, older adults have become the fastest-growing fraction of the world’s population, bringing a large number of social challenges to most countries [1–3]. In China, 254 million people were aged 60 years or over in 2019, accounting for almost 18.1% of the total population. The older population is increasing by approximately 6.2 million annually and is projected to reach 280 million by 2025, representing approximately one-fifth of China’s total population, which poses unprecedented challenges for the Chinese social care and health care systems [1]. The advanced ageing era comes at a price, and current social security and health care systems [4], particularly in developing countries, are not well prepared to address the financial burden [5–7]. Among older adults, centenarians are a relatively rare but growing group who have survived the diseases and disabilities that are common causes of death among non-centenarians and have attracted increasing concern [1, 8, 9].

Ageing is often associated with an increased incidence of health conditions, especially greater susceptibility to a loss of functional independence. Among centenarians, frailty, morbidity and a high rate of hospitalisation are common [6, 7]. A previous study using the 2005 Chinese Longitudinal Healthy Longevity Survey (CLHLS) data suggested that estimations of daily care needs, such as full-time care days needed, are necessary for policy-makers to identify the oldest-old with the greatest need for health care and social care services before death [10]. A population-based study of centenarians in China found that the prevalence of disabilities increases as death approaches, and disability rates are highest among centenarians compared with other age groups [1]. Another Chinese study found that centenarians experience substantial increases in death rates, disability scores in activities of daily living (ADLs) and cognitive impairment and a decrease in objective physical performance compared with their younger peers aged 80–99 years old [9]. However, several studies from high-income countries indicate that recent improvements in standards of living arrangements, health care technologies and the accessibility of health care services may lead to decreased rates of disability [9, 11]. One study in the United States demonstrated that as death approaches, centenarians tend to have later onset of serious diseases and a progressive compression of disability and morbidity compared with younger cohorts of oldest-old [12]. A Danish study found that the oldest-old of the 1915 cohort had better performance of cognitive functioning and ADLs than those in the 1905 cohort [1]. In other words, most members of the 1905 cohort survived life-threatening conditions but at the cost of poorer health [1, 9].

Medical expenditures, especially during the last years of life, are another inevitable financial burden as death approaches among the oldest-old population [13, 14]. A previous study indicated that medical expenditure during the last year of life occupied up to 10% of the entire lifespan health care cost [15]. Another study among the oldest-old in Japan revealed that end-of-life (EOL) medical expenditures increase as older adults age [16]. However, a population-based study from China found that individuals’ EOL medical expenditures did not differ significantly by age [17]. Another indicator to measure the quality of EOL care is the place of death, which is associated with the comfort experience of “a good death” [18]. Generally, most of older adults are cared for and die at their homes [19]. One previous study described a significant conversion in the place of death of the oldest-old [20]. A population-based study using the first three waves of the Chinese Longitudinal Healthy Longevity Survey (CLHLS) from 1998–2002 found that oldest-old individuals with higher socioeconomic status and worse health status were more likely to die at hospitals [20]. A population-based cohort study in the United Kingdom indicated that nursing homes or hospitals were the most common place of death (77%) [21]. A cross-sectional study in Japan showed that old adults wished to die at home, and the annual ratio of home deaths to all deaths was approximately 14% among people aged over 65 years [22]. This distinction between East Asian and Western countries may result from cultural and medical contexts [23]. However, in China, updated evidence on how older people’s place of death varies by patterns of age and to what extent is scarce [20, 24].

With the increasing number of oldest-old individuals in China, especially centenarians, access to quality EOL care is limited, which raises public concerns about how to provide efficient and sufficient EOL services for this population. Additionally, individual views about EOL care have changed dramatically in recent decades. An increasing number of people prioritise quality of life rather than considering extended survival as the primary outcome, giving rise to the reconsideration of what a “good death” is [25]. However, in mainland China, the EOL health care system has only recently launched and remains at an early stage with limited availability of palliative and hospice care services. Additionally, most current domestic studies centred on patients with end-stage cancer aim to provide comfort and control pain and other symptoms [16]. Variations in EOL medical expenditure, place of death and functional disability rates by age patterns among the oldest-old population have not been sufficiently investigated in China. Therefore, examining how these indicators vary within different age groups of the oldest-old population is important to provide novel clues concerning age-dependent EOL care services. Using data derived from the 1998–2018 CLHLS dataset, one of the largest samples of oldest-old individuals in the world, this study aims to compare the prevalence of functional disability in ADLs, place of death and medical expenditures during the last year of life between centenarians and non-centenarians in China.

## Methods

### Data source

**Fig. 1:**
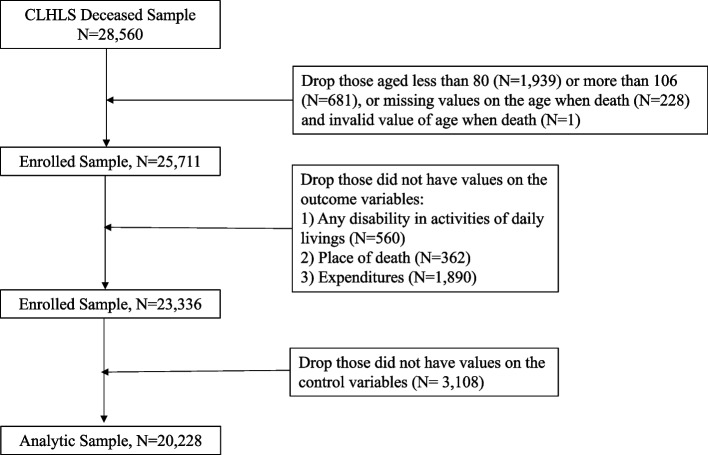
Derivation of analytic sample. Note: It is recommended to exclude respondents who were less than 80 years old if the analysis is focused on the oldest-old since they had not reached the oldest-old age (80 +) at the survey time. Weight is not applied to respondents aged less than 80 or more than 106 at their first interviews. CLHLS, Chinese Longitudinal Healthy Longevity Survey

As a nationally representative survey of the oldest-old population in China, the CLHLS aims to investigate the determinants of health longevity with three comparable samples of male and female centenarians, nonagenarians and octogenarians randomly selected from half of counties/cities across 23 provinces in China [9]. Starting in 1998, follow-up interviews and future recruitment of respondents were conducted in 2000–2018. A comprehensive set of individuals’ information was collected through in-home interviews, including sociodemographic and socioeconomic characteristics, health conditions, lifestyle, disability in ADLs, and availability of medical services. Written informed consent was obtained from each respondent or next-of-kin when the respondent was unable to write. Detailed information on the sampling design and other key information of the CLHLS is published elsewhere [8, 26]. The CLHLS was approved by the Research Ethics of Peking University and Duke University. Given the use of publicly available data, the protocol of the current study was reviewed and exempted by the Nanjing Medical University Institutional Review Board. Because weight was not applied to respondents aged less than 80 or more than 106 years old at their first interviews, 2,620 respondents were excluded from the current study. Respondents with no reports of any or valid values on the outcome variables and potential covariates were also excluded. Thus, a final sample of 20,228 decedents was analysed in this study (Fig. 1).

### Measures

The Katz index was used to assess disability in ADLs (being incontinent or needing assistance in performing one or more tasks of bathing, transferring, dressing, eating and toileting) before dying [26]. Its reliability and validity have been tested extensively [8, 26]. Following previous studies [9, 27], full dependence was defined as disability in the aforementioned six essential ADLs; partial dependence was defined as being incontinent or needing assistance in performing one or more of the five other ADLs; and full independence was defined as not being incontinent or needing assistance in performing one or more of the five other ADLs. Place of death was categorised into hospitals and others (home or nursing home facilities). Each respondent was asked about the *estimated total medical cost in the last year of the decedent’s life*. Our primary independent variable was the age of the oldest-old when they died, and the included oldest-old were categorised into three groups: 1) centenarians (≥ 100); 2) nonagenarians (90–99); and 3) octogenarians (80–89). Based on previous findings, the following factors were included as covariates: 1) sociodemographic and socioeconomic characteristics: rurality, gender, type of primary caregivers, years of schooling, number of children, annual per-capita household income, main source of income before dying, marital status [28], and previous white-collar job before retirement [5]; 2) living arrangements: alone, in a nursing home, with spouse only, living with other family members and timely medical services [29]; 3) health conditions: bedridden before dying, number of comorbidities [30]; and 4) census region, survey wave, and birth cohort [31].

### Statistical analysis

First, medical expenditures and household income were adjusted to the 2018 values using the consumer price index in the China Health Statistics Yearbook. Chi-square or Fisher exact tests were used to compare differences in individual characteristics by age group. Weighted logistics regression models were conducted to estimate the differences in the prevalence of disability in ADLs and place of death between centenarians, nonagenarians and octogenarians with other covariates controlled. Given that medical expenditure data did not have a normal distribution, a generalised linear model with a gamma distribution and log link function was used to estimate the association between EOL medical expenditures and age groups. Multicollinearity was assessed using the variance factor, and living alone and with a spouse only, survey wave and birth cohort were highly correlated (VIF > 5) [32]. The final models were selected based on the lowest values of the Akaike Information Criterion and Bayesian Information Criterion. The marginal differences were estimated to facilitate the estimation of diverse outcomes for defined groups with the change in the age patterns [33]. All procedures were carried out with Stata 15.0. A *P* value less than 0.05 was considered statistically significant.

## Results

### Individual characteristics of the included oldest-old

As shown in Table 1, among the 20,228 oldest-old, the decedents were 8,201 centenarians, 8,260 nonagenarians and 3,767 octogenarians. Over half (58.6%, weighted, hereafter) were female, and 67.3% resided in rural areas. Compared with younger oldest-old individuals, centenarians were more likely to be female (*P* < 0.001), live in urban areas (*P* < 0.001), die at their homes (*P* < 0.001), receive informal care (*P* = 0.005), be illiterate (*P* < 0.001), and be affluent (*P* < 0.001). Centenarians were less likely to have retirement wages as the main source of their income (*P* < 0.001), previously have white-collar jobs before retirement (*P* < 0.001), or have any chronic disease (*P* < 0.001). Summary statistics of the included samples by survey wave are presented in Additional file 1.

**Table 1:** Sample characteristics by age group

Characteristics	Overall	Age Group			Chi^2^	*P* value
	**(*N* = 20,228)**	**80–89 (*N* = 3,767)**	**90–99 (*N* = 8,260)**	**100–106 (*N* = 8,201)**		
	**Number (Weighted, Column %)**					
**Gender**					1471.6	< 0.001
Male	7691 (41.4)	2073 (44.0)	3755 (32.5)	1863 (27.6)		
Female	12537 (58.6)	1694 (56.0)	4505 (67.5)	6338 (72.4)		
**Place of death**					143.6	< 0.001
Home	18355 (87.5)	3236 (86.3)	7522 (91.6)	7597 (92.8)		
Hospital	1396 (8.8)	390 (9.6)	565 (6.2)	441 (5.0)		
Nursing Home	477 (3.7)	141 (4.1)	173 (2.2)	163 (2.2)		
**Primary caregivers** (informal)	19513 (95.0)	3581 (94.5)	8003 (96.8)	7929 (96.6)	27.2	< 0.001
**Rurality** (rural)	11975 (67.3)	2100 (68.2)	4832 (64.3)	5043 (58.4)	38.1	< 0.001
**Years of schooling**					927.9	< 0.001
0	14791 (67.4)	2216 (65.1)	5709 (75.1)	6866 (80.9)		
≥ 1	5437 (32.6)	1551 (34.9)	2551 (24.9)	1335 (19.1)		
**Number of children even born**					8.4	0.079
0–2	4158 (20.4)	730 (20.0)	1758 (22.0)	1670 (21.1)		
3–4	5888 (29.0)	1118 (29.2)	2421 (28.3)	2349 (27.7)		
> 4	10182 (50.6)	1919 (50.8)	4081 (49.7)	4182 (51.3)		
**Per capita household income annually** ^a^					41.4	< 0.001
< 2,673	5003 (35.3)	997 (36.3)	1989 (32.1)	2017 (26.2)		
2,673–6,596	5177 (30.5)	984 (30.9)	1993 (29.0)	2200 (27.9)		
6,596–21,420	5017 (20.1)	950 (19.9)	2085 (20.8)	1982 (21.8)		
> 21,420	5031 (14.1)	836 (12.9)	2193 (18.1)	2002 (24.2)		
**Main Source of Income**						
Retirement wage	1983 (11.2)	588 (12.0)	938 (8.3)	457 (6.4)	332.1	< 0.001
Family members	16920 (82.5)	2914 (81.5)	6847 (86.0)	7159 (86.0)	192.2	< 0.001
**Being married during the last year of Life**	17802 (20.3)	2740 (23.1)	7200 (11.0)	7862 (4.7)	1317.7	< 0.001
**Living arrangement**						
Alone	2018 (11.3)	469 (11.5)	914 (10.9)	635 (7.8)	82.1	< 0.001
In nursing homes	489 (4.4)	165 (5.0)	174 (2.4)	150 (2.2)	76.9	< 0.001
With spouse only	15418 (12.7)	2487 (13.1)	6146 (11.2)	6785 (13.0)	423.2	< 0.001
With other family members	1957 (70.2)	596 (69.1)	882 (74.1)	479 (75.4)	310.3	< 0.001
**Had white-collar job before retirement**	822 (4.7)	264 (5.1)	381 (3.4)	177 (2.4)	166.5	< 0.001
**Number of comorbidities**					208.3	< 0.001
0	7288 (33.1)	1066 (31.7)	3012 (37.8)	3210 (40.5)		
1	6478 (32.8)	1198 (32.7)	2612 (33.3)	2668 (30.8)		
2	2937 (15.2)	626 (15.6)	1214 (13.8)	1097 (14.0)		
≥ 3	3525 (18.9)	877 (20.0)	1422 (15.1)	1226 (14.6)		
**Bedridden before dying**	15003 (72.9)	2836 (72.8)	6081 (73.5)	6086 (73.7)	3.8	0.153
**Timely medical services**					266.6	< 0.001
No	15122 (81.6)	3106 (82.9)	6237 (77.6)	5779 (70.1)		
Yes	1328 (8.1)	266 (8.2)	515 (7.7)	547 (9.1)		
Was not ill	3778 (10.3)	395 (8.9)	1508 (14.8)	1875 (20.8)		
**Region**					57.2	< 0.001
Eastern	7756 (35.7)	1321 (35.2)	3133 (37.1)	3302 (41.3)		
Central	4705 (24.4)	923 (24.3)	1991 (25.0)	1791 (21.5)		
Western	6176 (31.5)	1182 (31.5)	2574 (31.4)	2420 (29.6)		
Northeast	1591 (8.5)	341 (9.0)	562 (6.5)	688 (7.6)		
**Cohort**					15074.7	< 0.001
Cohort 1891–1900	3402 (0.4)	0 (0)	153 (0.7)	3249 (34.1)		
Cohort 1901–1910	7864 (13.3)	104 (2.8)	3337 (50.2)	4423 (43.0)		
Cohort 1911–1920	6945 (61.1)	2184 (68.2)	4234 (36.6)	527 (22.8)		
Cohort 1921–1930	1873 (22.4)	1336 (25.4)	536 (12.5)	1 (0)		
Cohort 1831–1940	144(2.8)	143 (3.6)	0 (0)	1 (0)		

Values are represented as number (percentage) unless otherwise indicated. Numbers were calculated from study samples (unweighted). Percentages were calculated using the age-sex-rural/urban-specific sample weights. a, presented in Chinese yuan (1 Chinese yuan = 0.15 US dollars); b, divorced, widowed, and never married. *ADLs* activities of daily living

### Prevalence of disability, place of death and EOL medical expenditures between centenarians and younger oldest-old

As shown in Table 2, after controlling for other covariates, centenarians and nonagenarians had greater probabilities of full dependence (average marginal difference, 95% CI: nonagenarians vs. octogenarians: 2.7% [0%, 5.3%]; centenarians vs. octogenarians: 3.8% [0.3%, 7.9%]) and partial dependence (nonagenarians vs. octogenarians: 6.9% [3.4%, 10.3%]; centenarians vs. octogenarians: 15.1% [10.5%, 19.8%]) than octogenarians. Substantial decreases were observed in the prevalence of full independence with increasing age (nonagenarians vs. octogenarians: –8.9% [–11.6%, –6.2%]; centenarians vs. octogenarians: –16.0% [–19.1%, –12.8%]; centenarians vs. nonagenarians: –7.0% [–9.2%, –4.9%]). In Table 3, nonagenarians reported a smaller prevalence of death in hospitals than octogenarians (–3.0% [–4.7%, –1.2%]), and the downwards trend continued among centenarians (–4.3% [–6.3%, –2.2%]). However, differences in EOL medical expenditures during the last year of life between nonagenarians, centenarians and octogenarians were not statistically significant (Table 4).

**Table 2:** Differences in disability in ADLs between centenarians and younger oldest-old

	**Full dependence**	**Partial dependence**	**Full independence**
	Average Marginal Differences (%, 95% CI)		
**Nonagenarians vs. octogenarians**	2.7 (0, 5.3)*	6.9 (3.4, 10.3)***	-8.9 (-11.6, -6.2)***
**Centenarians vs. octogenarians**	3.8 (0.3, 7.9)*	15.1 (10.5, 19.8)***	-16.0 (-19.1, -12.8)***
**Centenarians vs. nonagenarians**	1.1 (-2.0, 4.2)	8.2 (5.0, 11.6) ***	-7.0 (-9.2, -4.9) ***

Gender, residence rurality, type of primary caregivers, years of schooling, per capita household income, main source of income before dying, being married during the last year of life or not, having a white-collar job before retirement or not, living arrangement, timely medical services, census region and birth cohort were set as covariates. *CI* confidence interval. ADLs, activities of daily living

*, *P* < 0.05; ***, *P* < 0.001

**Table 3:** Differences in death in the hospitals between centenarians and younger oldest-old

	**Death in Hospitals**
	Average Marginal Differences (%, 95% CI)
**Nonagenarians vs. octogenarians**	-3.0 (-4.7, -1.2)**
**Centenarians vs. octogenarians**	-4.3 (-6.3, -2.2)***
**Centenarians vs. nonagenarians**	-1.3 (-2.5, -0.1)*

Gender, residence rurality, type of primary caregivers, years of schooling, per capita household income, main source of income before dying, being married during the last year of life or not, having a white-collar job before retirement or not, living arrangement, being bedridden before dying or not, timely medical services, census region and birth cohort were set as covariates. *, *P* < 0.05, **, *P* < 0.01; ***, *P* < 0.001

**Table 4:** Marginal differences in the EOL medical expenditure during the Last year of Life between centenarians and younger oldest-old

	**EOL medical expenditures**
	Average Marginal Differences (95% CI)
**Nonagenarians vs. octogenarians**	606 (-290, 1,503)
**Centenarians vs. octogenarians**	401 (-894, 1,697)
**Centenarians vs. nonagenarians**	-205 (-1,096, 686)

Gender, residence rurality, type of primary caregivers, years of schooling, per capita household income, main source of income before dying, being married during the last year of life or not, having a white-collar job before retirement or not, living arrangement, being bedridden, timely medical services, census region and birth cohort were set as covariates. a, presented in Chinese yuan (1 Chinese yuan = 0.15 US dollars)

In the sensitivity analysis, we used subgroups that reported missing values of the covariates as comparison groups in the regression models. As shown in Additional file 1, centenarians and nonagenarians had a greater likelihood of full dependence (nonagenarians vs. octogenarians: 3.3% [0.9%, 5.8%]; centenarians vs. octogenarians: 4.8% [1.0%, 8.6%]) and partial dependence (nonagenarians vs. octogenarians: 6.4% [3.2%, 9.5%]; centenarians vs. octogenarians: 14.1% [9.7%, 18.4%]) than octogenarians. Substantial decreases were also observed in the prevalence of full independence with increasing age. Moreover, as shown in Additional file 1, centenarians and nonagenarians reported a smaller likelihood of death in hospitals than octogenarians (nonagenarians vs. octogenarians: -2.7% [-4.3%, -1.0%]; centenarians vs. octogenarians: -4.2% [-6.1%, -2.4%]). In addition, as shown in Additional file 1, nonagenarians had more EOL medical expenditures than octogenarians (Chinese Yuan: 908 [95, 1,723]). The directions and significance of the main results estimated by the sensitivity analysis are consistent with the above results.

## Discussion

In this study, disparities in the functional disabilities in ADLs before dying and the place of death between centenarians and non-centenarians were observed. However, no statistically significant differences in EOL medical expenditure during the last year of life were observed. Our findings may enrich the current literature on EOL care services for centenarians and their younger peers, which highlights the importance of optimising the provision of long-term and EOL care by age patterns. Future policy efforts should be enacted beyond medical care systems to make resource allocation more efficient to promote successful ageing for the oldest-old in China.

First, the prevalence of full and partial dependence in ADLs rises substantially as age increases, which is in accordance with natural ageing and indicates a greater likelihood of frailty among nonagenarians and centenarians [34]. This result suggests that an increasing proportion of oldest-old individuals need external assistance in ADLs [5]. Previous studies have suggested that positive survival selection may result in such a decrease in the prevalence of full dependence on ADLs; that is, older adults with full dependence have limited chances of living longer, and older adults who survive to become centenarians are more healthy than expected [35]. A study in the United States supports these findings that most older adults who achieve exceptional longevity are in relatively good health and do not have significant loss of daily functions [12]. Many of the oldest-old population appear to be more tolerant to certain pathologies, showing fewer body symptoms and obstacles than expected by medical pathological examination [36]. Furthermore, in Germany, individuals who died as centenarians had fewer comorbidities than their younger peers [37]. Our results suggest that the prevalence of full independence in ADLs declined as age increased after the birth cohort and other covariates were adjusted, which is similar to findings of the China Hainan Centenarian Cohort Study that centenarians had a significantly higher prevalence of cognitive decline than other oldest-old adults [38]. Additionally, high scores of subjective well-being would lead to exceptional longevity [39]. Although successive generations of Chinese oldest-old people are living with less disability [1], a greater prevalence of full independence in ADLs means higher daily care service needs among nonagenarians and centenarians. Therefore, policy-makers should pay more attention to populations that are living longer to meet their daily care service needs by allocating adequate long-term care and community care services to the oldest-old of differential health status in appropriate health care settings.

Second, compared with non-centenarians, centenarians are less likely to die in hospitals. This tendency is similar to the findings of another Chinese study [24]. As causes of death are highly correlated with illness trajectories and change with increasing age, this result can be partially explained by the different patterns of causes of death [40]. Centenarians are less likely to have chronic diseases as causes of death than their younger peers [35] but are more likely to suffer from pneumonia and ischaemic heart disease, acute deterioration in health status, a relatively rapid terminal decline and greater risk of dying in a short time before receiving care in hospitals [40]. These tendencies partially explain the findings that centenarians experience fewer hospitalisations [34]. Moreover, as more older adults tend to receive care in nursing homes, the increasing proportion of deaths in nursing homes may be another reason [41]. Although many of the oldest-old population have their own thoughts about their place of death, a gap remains between the place where older adults wish to die and the actual place of death [25]. The traditional Chinese cultural concept that “fallen leaves return to the root” might cause older adults to want to die at their homes, which would provide greater physical and emotional comfort and a sense of belonging. Therefore, most older adults in China prefer to receive their EOL care at home. Additionally, support from their children or grandchildren could increase the feasibility of dying at their preferred place by providing more informal care [42]. However, filial piety might push their children to seek help from hospitals due to a lack of accessible hospice care [16], which might produce a high likelihood of medicalization of death. Therefore, cultural differences, preferences, values, and informal care support should be jointly considered. The provision of culturally appropriate long-term and EOL care facilities with adequate staffing may prevent unnecessary hospitalisations and deaths in hospitals among the oldest-old population in China.

Third, after controlling for birth cohort and other covariates, our study revealed that differences in EOL medical expenditures between centenarians, octogenarians and nonagenarians were not statistically significant. These findings are consistent with findings from one population-based study from China showing that individuals’ EOL medical expenditures did not increase significantly as age increased [18]. However, the oldest-old in Japan spent more on EOL medical expenditures as their age increased [17]. These results might be related to the fact that centenarians experienced fewer hospitalisations than their younger counterparts [34, 35]. Although older adults often suffer from multiple chronic diseases [36] and survive at the cost of poorer health, previous studies have found that centenarians prefer to die at home, where they are more likely to feel cosy and comfortable, rather than being treated meaninglessly in hospitals [43]. These results might be related to the fact that centenarians did not report a greater number of comorbidities with advanced ageing; familial and societal attitudes towards the intensity of EOL care for oldest-old individuals might have changed with social development [43–47]. Therefore, future policy design, such as health insurance policies for centenarians and their younger peers, should go beyond medical care services to support people living with extreme longevity.

The large samples in the CLHLS were recruited nationally, which can prevent bias from regional research to a certain extent. However, this study still has several limitations. First, the assessment of disability among the oldest-old is only restricted to ADL disability, and instrumental ADL is not included. This reliance on a single measure of disability may restrict our findings to other aspects of health status. Second, the oldest-old in our study were prone to recall and incomplete information biases, especially on the cost data. Additionally, the CLHLS survey obtained disability assessments based on proxy reports, but the usage of the proxy method is considered appropriate to omit observations to a certain extent when the oldest-old are unable to respond to investigators. Third, a total of 2,342 oldest-old individuals did not report any values on the outcome variables. Compared with oldest-old reported outcome variables, these oldest-old individuals reported no values on the outcome variables tended to be younger, more likely to live in urban areas and to be female (Additional file 1). Fourth, the current study focused on the death sample in the last year of life. Future research should compare ADLs and medical expenditures among oldest-old survivors to improve policy implementation.

## Conclusions

In this national population-based study of the oldest-old population, the prevalence of full and partial dependence in ADLs increased with increasing age, and the prevalence of full independence in ADLs decreased. The probabilities of hospital deaths tended to be lower for centenarians in China than for their younger peers, while EOL medical expenditures did not differ by age group. Therefore, future policy efforts, including financial incentives to enhance community social care resources for the largest oldest-old population in the world, are warranted.

## Supplementary Information

**Additional file 1: Appendix Table 1.** Summary statistics of the included Chinese longitudinal health longevity survey samples by survey wave. **Appendix Table 2.** Differences in disability in ADLs by age group (*N*=23336). **Appendix Table 3.** Differences in death in the hospitals by age group (*N*=23336). **Appendix Table 4.** Marginal differences in the EOL medical expenditure during the last year of Life by age group (*N*=23336). **Appendix Table 5.** Comparison of basic characteristics between oldest-old reported and not reported outcome variables data

## Data Availability

All the data are available from Peking University Open Research Data. Centre for Healthy Ageing and Development Studies, 2020, "The Chinese Longitudinal Healthy Longevity Survey (CLHLS)-Longitudinal Data (1998–2018). https://doi.org/10.18170/DVN/WBO7LK, Peking University Open Research Data Platform, V2.

## Abbreviations

EOL: End of life
CLHLS: Chinese Longitudinal Health Longevity Survey
ADLs: Activities of daily living
CI: Confidence interval
