# Supplementary material for: Comparison of functional disabilities, place of death and end-of-life medical expenditures among centenarians and non-centenarians in China: a series of cross-sectional studies
Source: BMC Geriatr. 2023 Jun 30;23:402. doi: 10.1186/s12877-023-04111-w (PMC10311848; doi:10.1186/s12877-023-04111-w)
Supplement: Supplementary file 1 — Additional file 1: Appendix Table 1. Summary statistics of the included Chinese longitudinal health longevity survey samples by survey wave. Appendix Table 2. Differences in disability in ADLs by age group (N=23336). Appendix Table 3. Differences in death in the hospitals by age group (N=23336). Appendix Table 4. Marginal differences in the EOL medical expenditure during the last year of Life by age group (N=23336). Appendix Table 5. Comparison of basic characteristics between oldest-old reported and not reported outcome variables data [file 12877_2023_4111_MOESM1_ESM.docx]

# Appendix Table 1. Summary Statistics of the Included Chinese Longitudinal Health Longevity Survey Samples by Survey Wave

| **Characteristic** | **1998 (N=2,410)** | **2000 (N=2,702)** | **2002 (N=4,160)** | **2005 (N=3,687)** | **2008/2009 (N=4,091)** | **2011/2012 (N=1,827)** | **2014 (N=1,351)** | **Chi^2^** | ***P*** |
| --- | --- | --- | --- | --- | --- | --- | --- | --- | --- |
|  | **Number (Weighted, Column %)** | | | | | | |  |  |
| **Gender** |  |  |  |  |  |  |  | 20.1 | 0.003 |
| Male | 897 (41.0) | 1,041 (40.3) | 1,544 (42.7) | 1,387 (42.6) | 1,510 (45.5) | 741 (44.7) | 571 (45.5) |  |  |
| Female | 1,513 (59.0) | 1,661 (59.7) | 2,616 (57.3) | 2,300 (57.4) | 2,581 (54.5) | 1,086 (55.3) | 780 (54.5) |  |  |
| **Place of death** |  |  |  |  |  |  |  | 52.5 | < 0.001 |
| Home | 2,216 (86.9) | 2,405 (85.8) | 3,755 (88.1) | 3,312 (90.7) | 3,752 (89.4) | 1,674 (91.9) | 1,241 (89.4) |  |  |
| Hospital | 132 (8.8) | 219 (9.5) | 275 (10.0) | 296 (7.7) | 275 (7.7) | 113 (6.9) | 86 (7.7) |  |  |
| Nursing Home | 62 (4.3) | 78 (4.7) | 130 (1.9) | 79 (1.6) | 64 (2.9) | 40 (1.3) | 24 (2.9) |  |  |
| **Primary caregivers** (informal) | 2,311 (93.9) | 2,561 (93.2) | 3,907 (98.1) | 3,602 (98.2) | 4,019 (97.2) | 1,785 (98.9) | 1,328 (97.2) | 179.5 | < 0.001 |
| **Rurality** (rural) | 1,656 (67.4) | 1,154 (69.4) | 2459 (63.0) | 2,210 (69.4) | 2,788 (60.5) | 917 (58.4) | 791 (60.5) |  | < 0.001 |
| **Years of schooling** |  |  |  |  |  |  |  | 39.8 | < 0.001 |
| 0 | 1,811 (68.0) | 1,918 (69.6) | 2,991 (66.3) | 2,697 (67.7) | 3,113 (63.0) | 1,310 (64.1) | 951 (63.0) |  |  |
| ≥1 | 599 (32.0) | 784 (30.4) | 1,169 (33.7) | 990 (32.3) | 978 (37.0) | 517 (35.9) | 400 (37.0) |  |  |
| **Number of children even born** |  |  |  |  |  |  |  | 22.4 | 0.033 |
| 0-2 | 501 (20.4) | 593 (24.3) | 906 (18.9) | 763 (16.5) | 796 (14.6) | 350 (15.1) | 249 (14.6) |  |  |
| 3-4 | 654 (26.4) | 762 (28.6) | 1,207 (29.0) | 1,091 (31.8) | 1,213 (32.0) | 555 (28.9) | 406 (32.0) |  |  |
| > 4 | 1,255 (53.3) | 1,347 (47.1) | 2,047 (52.1) | 1,833 (51.7) | 2,082 (53.4) | 922 (56.0) | 696 (53.4) |  |  |
| **Per capita household income annually** |  |  |  |  |  |  |  | 6086.4 | < 0.001 |
| < 2,673 | 1,046 (43.5) | 1,153 (48.2) | 1,462 (10.6) | 336 (14.6) | 540 (18.0) | 263 (16.1) | 203 (18.0) |  |  |
| 2,673-6,596 | 1,018 (41.8) | 950 (34.0) | 1,505 (14.3) | 519 (13.9) | 633 (19.3) | 309 (17.3) | 243 (19.3) |  |  |
| 6,596-21,420 | 304 (13.1) | 524 (16.7) | 943 (40.5) | 1,404 (28.2) | 1,069 (24.6) | 417 (22.1) | 356 (24.6) |  |  |
| > 21,420 | 42 (1.6) | 75 (1.1) | 250 (34.6) | 1,428 (43.3) | 1,849 (38.1) | 838 (44.5) | 549 (38.1) |  |  |
| **Main Source of Income** |  |  |  |  |  |  |  |  |  |
| Retirement wage | 227 (13.1) | 254 (9.1) | 363 (12.0) | 374 (10.2) | 363 (16.8) | 212 (13.0) | 190 (16.8) | 45.3 | < 0.001 |
| Family members | 2,029 (80.7) | 2,292 (85.3) | 3,518 (84.9) | 3,160 (82.2) | 3,465 (74.0) | 1,434 (78.6) | 1,022 (74) | 119.3 | < 0.001 |
| **Being married during the**  **last year of life** | 198 (19.0) | 255 (16.3) | 457 (20.5) | 359 (26.3) | 608 (29.6) | 296 (28.5) | 253 (29.6) | 191.7 | < 0.001 |
| **Living alone** | 165 (7.7) | 251 (10.7) | 336 (13.9) | 446 (10.8) | 358 (28.9) | 181 (11.8) | 281 (28.9) | 245.9 | < 0.001 |
| **Once had white-collar jobs before retirement** | 84 (5.8) | 117 (3.2) | 177 (7.1) | 165 (5) | 149 (5.3) | 70 (4.1) | 60 (5.3) | 7.2 | 0.306 |
| **Number of comorbidities** |  |  |  |  |  |  |  | 3146.7 | < 0.001 |
| 0 | 1,073 (44.4) | 859 (28.6) | 217 (35.6) | 1,468 (47.2) | 2,307 (34.8) | 780 (31.1) | 584 (34.8) |  |  |
| 1 | 883 (37.1) | 967 (33.7) | 1,856 (22.8) | 886 (30.3) | 1,081 (25.5) | 472 (26.4) | 333 (25.5) |  |  |
| 2 | 297 (11.1) | 374 (15.8) | 951 (15.0) | 454 (13.7) | 466 (16.2) | 223 (14.2) | 172 (16.2) |  |  |
| ≥3 | 157 (7.4) | 502 (21.9) | 1,136 (26.6) | 879 (8.8) | 237 (23.5) | 352 (28.3) | 262 (23.5) |  |  |
| **Bedridden before dying** | 1,728 (68.4) | 1,957 (71.3) | 3,194 (72.9) | 2,667 (77.7) | 3,040 (77.2) | 1,396 (78.1) | 1,021 (77.2) | 39.4 | < 0.001 |
| **Timely medical services** |  |  |  |  |  |  |  | 1946.3 | < 0.001 |
| No | 72 (3.9) | 603 (14.5) | 221 (6.4) | 163 (4.8) | 172 (4.0) | 60 (4.7) | 37 (4.0) |  |  |
| Yes | 1,661 (76.9) | 2,099 (85.5) | 2,976 (84.1) | 2,940 (84.0) | 3,090 (82.5) | 1,341 (82.1) | 1,015 (82.5) |  |  |
| Was not ill | 677 (19.2) | 0 (0) | 963 (9.5) | 584 (11.2) | 829 (13.5) | 426 (13.2) | 299 (13.5) |  |  |
| **Region** |  |  |  |  |  |  |  | 314.5 | < 0.001 |
| Eastern | 980 (36.1) | 1,059 (35.8) | 1,574 (33.6) | 1,188 (29.7) | 1,550 (31.5) | 731 (37.5) | 674 (50.3) |  |  |
| Central | 447 (20.0) | 543 (25.7) | 877 (20.8) | 993 (30.5) | 1,120 (30.9) | 414 (24.4) | 311 (22.3) |  |  |
| Western | 789 (34.3) | 845 (29.4) | 1,304 (34.3) | 1,191 (31.4) | 1,141 (31.6) | 567 (32.0) | 339 (25.6) |  |  |
| Northeast | 194 (9.6) | 255 (9.2) | 405 (11.3) | 315 (8.7) | 280 (6.1) | 115 (6.1) | 27 (1.8) |  |  |
| **Cohort** |  |  |  |  |  |  |  | 9430.4 | < 0.001 |
| Cohort 1891-1900 | 1,256 (1.1) | 966 (0.4) | 938 (0) | 242 (0) | 0 (0) | 0 (0) | 0 (0) |  |  |
| Cohort 1901-1910 | 820 (29.0) | 1,024 (14.7) | 1,946 (3.5) | 1,804 (1.3) | 1,742 (0) | 428 (0.3) | 100 (0) |  |  |
| Cohort 1911-1920 | 334 (69.9) | 712 (84.9) | 1,187 (49.9) | 1,466 (26.8) | 1,774 (7.5) | 883 (14.2) | 589 (7.5) |  |  |
| Cohort 1921-1930 | 0 (0) | 0 (0) | 89 (46.6) | 174 (71.9) | 575 (58.5) | 490 (75.7) | 545 (58.5) |  |  |
| Cohort 1831-1940 | 0 (0) | 0 (0) | 0 (0) | 1 (0) | 0 (34.0) | 26 (9.8) | 117 (34.0) |  |  |

Note: Values were represented as No. (percentages) unless otherwise indicated. No, was calculated from study samples (unweighted). Percentages were calculated using the age-sex-rural/urban-specific sample weights. a, presented in Chinese Yuan (1 Chinese Yuan=0.15 US dollars); b, divorce, widowed, and never married. ADLs, activities of daily livings. As this study focus on the deceased oldest-old, most of data of covariates were collected in the 1998-2014 survey.

# Appendix Table 2. Differences in Disability in ADLs by Age Group (N=23336)

|  | **Full dependence** | **Partial dependence** | **Full independence** |
| --- | --- | --- | --- |
|  | Average Marginal Differences (%, 95% CI) | | |
| **Nonagenarians vs. octogenarians** | 3.3 (0.9, 5.8)** | 6.4 (3.2, 9.5)*** | -8.9 (-11.4, -6.4)*** |
| **Centenarians vs. octogenarians** | 4.8 (1.0, 8.6)* | 14.1 (9.7, 18.4)*** | -15.8 (-18.8, -12.7)*** |
| **Centenarians vs. nonagenarians** | 1.4 (-1.3, 4.3) | 7.7 (4.6, 10.8) *** | -6.9 (-8.9, -4.7) *** |

Note: gender, residence rurality, type of primary caregivers, years of schooling, per capital household income, main source of income before dying, being married during the last year of life or not, once had white-collar job before retirement or not, living arrangement, timely medical services, census region and birth cohort were set as covariates. CI, Confidence interval. ADLs, activities of daily living. *, *P* < 0.05, **, *P* < 0.01; ***, *P* < 0.001.

# Appendix Table 3. Differences in Death in the Hospitals by Age Group (N=23336)

|  | **Death in hospitals** |
| --- | --- |
|  | Average Marginal Differences (%, 95% CI) |
| **Nonagenarians vs. octogenarians** | -2.7 (-4.3, -1.0)** |
| **Centenarians vs. octogenarians** | -4.2 (-6.1, -2.4)*** |
| **Centenarians vs. nonagenarians** | -1.6 (-2.7, -0.4)** |

Note: gender, residence rurality, type of primary caregivers, years of schooling, per capital household income, main source of income before dying, being married during the last year of life or not, once had white-collar job before retirement or not, living arrangement, being bedridden before dying or not, timely medical services, census region and birth cohort were set as covariates. *, *P* < 0.05, **, *P* < 0.01; ***, *P* < 0.001.

# Appendix Table 4. Marginal differences in the EOL Medical Expenditure during the Last Year of Life by Age Group (N=23336)

|  | **EOL Medical expenditures** |
| --- | --- |
|  | Average Marginal Differences (95% CI) |
| **Nonagenarians vs. octogenarians** | 908 (95,1,723) * |
| **Centenarians vs. octogenarians** | 1,132 (-72, 2,338) |
| **Centenarians vs. nonagenarians** | 223 (-635, 1,082) |

Note: gender, residence rurality, type of primary caregivers, years of schooling, per capital household income, main source of income before dying, being married during the last year of life or not, once had white-collar job before retirement or not, living arrangement, being bedridden, timely medical services, census region and birth cohort were set as covariates. a, presented in Chinese Yuan (1 Chinese Yuan=0.15 US dollars). *, *P* < 0.05, **, *P* < 0.01; ***, *P* < 0.001.

# Appendix Table 5. Comparison of Basic Characteristics Between Oldest-old Reported and not Reported Outcome Variables Data

| **Characteristics** | **Overall** | **Oldest-old reported outcome variables (N=23,369)** | **Oldest-old did not reported outcome variables (N=2,342)** | **Chi2** | ***P*** |
| --- | --- | --- | --- | --- | --- |
| **Age at Death** |  | **Number (Weighted, Column %)** | | 6.5 | 0.039 |
| 80-89 | 4,927 | 4,432 (78.0) | 495 (79.8) |  |  |
| 90-99 | 10,524 | 9,587 (21.2) | 937 (19.4) |  |  |
| 100-106 | 10,260 | 9,350 (0.8) | 910 (0.8) |  |  |
| **Residence rurality** |  |  |  | 42.5 | < 0.001 |
| Urban | 10,723 | 9,598 (33.2) | 1,125 (40.3) |  |  |
| Rural | 14,988 | 13,771 (66.8) | 1,217 (59.7) |  |  |
| **Gender** |  |  |  | 5.9 | 0.016 |
| Female | 15,682 | 14,308 (57.8) | 1,374 (53.8) |  |  |
| Male | 10,029 | 9,061(42.2) | 968 (46.2) |  |  |
